# Electrical Stimulation at the ST36 Acupoint Protects against Sepsis Lethality and Reduces Serum TNF Levels through Vagus Nerve- and Catecholamine-Dependent Mechanisms

**DOI:** 10.1155/2014/451674

**Published:** 2014-06-26

**Authors:** Albino Villegas-Bastida, Rafael Torres-Rosas, Lourdes Andrea Arriaga-Pizano, Javier Flores-Estrada, Altamirano Gustavo-Acosta, Mario Adan Moreno-Eutimio

**Affiliations:** ^1^Medical Research Unit on Immunochemistry, Specialties Hospital, National Medical Centre “Siglo XXI,” Mexican Social Security Institute (IMSS), 06720 Mexico City, DF, Mexico; ^2^Universidad Autónoma Benito Juarez de Oaxaca (UABJO), 68120 Oaxaca de Juárez, OAX, Mexico; ^3^National School of Medicine and Homeopathy, National Polytechnic Institute, 07320 Mexico City, DF, Mexico; ^4^Immunobiology Laboratory, Mexico's Juarez Hospital, Ministry of Health, 07760 Mexico City, DF, Mexico

## Abstract

Electrical vagus nerve (VN) stimulation during sepsis attenuates tumor necrosis factor (TNF) production through the cholinergic anti-inflammatory pathway, which depends on the integrity of the VN and catecholamine production. To characterize the effect of electroacupuncture at ST36 (EA-ST36) on serum TNF, IL-6, nitrite, and HMGB1 levels and survival rates, based on VN integrity and catecholamine production, a sepsis model was induced in rats using cecal ligation and puncture (CLP). The septic rats were subsequently treated with EA-ST36 (CLP+ST36), and serum samples were collected and analyzed for cytokines levels. The serum TNF, IL-6, nitrite, and HMGB1 levels in the CLP+ST36 group were significantly lower compared with the group without treatment, the survival rates were significantly higher (*P* < 0.05), and the acute organ injury induced by CLP was mitigated by EA-ST36; however, when subdiaphragmatic vagotomy was performed, the serum levels of TNF in the CLP+ST36 group did not show a significant difference compared with the group without electrostimulation, and, similarly, no significant difference in serum TNF levels was found under the pharmacological blockade of catecholamines. These results suggest that in rats with CLP sepsis models EA-ST36 reduces serum TNF levels through VN- and atecholamine-dependent mechanisms.

## 1. Introduction

Acupuncture has been used for over 4,000 years and has recently experienced widespread use worldwide, with endorsements from the United States National Institutes of Health, the National Center for Complementary and Alternative Medicine, and the World Health Organization. Nevertheless, it has been difficult to establish a biological basis for acupuncture, due to the diversity of clinical practices related to this procedure, the lack of adequate clinical trials, and the diverse backgrounds of acupuncturists [1, 2]. Recently, acupuncture has been described as a complementary and alternative medicine (CAM) in which filiform needles are inserted at specific points on the body, called acupoints, which can subsequently be stimulated in various ways, such as through electroacupuncture (EA) [1]. Immunomodulatory effects have been reported after acupoint stimulation. Anti-inflammatory effects have been reported in mouse models of inflammation associated with EA at the Zusanli acupoint (ST36). Indeed, Gu et al. [3] reported that treatment with EA at ST36 induced a nephroprotective effect associated with decreased levels of TNF-*α* and interleukin-1 (IL-1) in a lipopolysaccharide-induced model of acute nephritis; Yim et al. reported that EA at ST36 decreases the TNF-*α* and IL-6 levels in a collagen-induced arthritis mouse model [4]; Wang et al. confirmed the reduction of TNF-*α* levels after ST36 stimulation in an ulcerative colitis rat model [5]; and Chae et al. observed that ST36 stimulation decreases proinflammatory cytokine expression in a carrageenan-induced mouse model of inflammation [6]. Recent studies have shown that EA at ST36 decreases the levels of TNF-*α*, IL-1-*β*, and IL-6 through the suppression of the Toll-like receptor 4 and nuclear factor-kappa B (TLR4/NF-*κ*B) signaling pathway in cerebral ischemia-reperfusion injured rats [7], while other studies have shown a reduction in NF-*κ*B DNA-binding activity in a passive cutaneous anaphylaxis model through EA stimulation at the ST36 acupoint [8].

Although acupuncture has been widely applied to treat inflammatory diseases, particularly in animal models [5–8], the precise mechanism underlying the effects of this treatment remains unknown.

Furthermore, TNF levels diminish with VN electrostimulation through the cholinergic anti-inflammatory pathway, involving catecholamine expression and the VN [9]. Thus, in the present study, we used cecal ligation and puncture (CLP) in rats to determine whether the anti-inflammatory effect of EA at ST36 depends on the anatomical integrity of the VN and catecholamine production in a sepsis model.

## 2. Materials and Methods

All experiments were performed in accordance with the approved animal protocols and guidelines established through the Mexican Social Security Institute (IMSS) National Scientific Research Commission and the international guidelines for the use and care of laboratory animals [10].

### 2.1. Animals

Male Wistar rats were obtained from the Experimental Medicine Department, Faculty of Medicine, National Autonomous University of Mexico (UNAM). A total of 490 rats (200–250 g) were used in this study and housed in standard plastic cages on sawdust bedding in an air-conditioned room at 22 ± 1°C. Standard rat food and tap water were provided ad libitum.

### 2.2. Sepsis Model

A CLP polymicrobial sepsis model was established in rats through cecal ligation and puncture. The rats were anesthetized through the intraperitoneal (i.p.) administration of 100 mg/kg of ketamine (Anesket, PiSA, Mexico) and 20 mg/kg of xylazine (Procin, PiSA, Mexico). The abdomen was shaved, and a midline incision was performed in the abdomen. Subsequently, the peritoneum was opened, and the cecum was isolated and ligated with a 3-0 Nylon (Nylon, Atramat, Mexico) ligature just proximal to the ileocecal valve (high-grade sepsis) [11]. Two punctures were made into the cecum on one side and through the cecal wall on the opposite side using a 21-gauge needle (Becton Dickinson, CA, USA) at 5 mm distal to the point of ligation. Subsequently, the stool was extruded (3 mm), the cecum was returned to the normal intra-abdominal position, and the abdomen was closed with a running suture of 3-0 sterile Nylon. The sham-operated group received laparotomies, and the rat cecum was manipulated, but not ligated or perforated. The operated animals were hydrated through the injection of prewarmed sterile isotonic saline (37°C; 5 mL per 100 g body weight) subcutaneously. Blood samples were obtained through cardiac puncture at two, six, and eighteen hours after CLP in independent assays.

### 2.3. Acupuncture Treatment Procedure

Two pairs of stainless steel needles (diameter, 0.3 mm; length, 30 mm (HBW Silver Star, HBW Supply Inc., CA, USA)) were inserted perpendicularly at a depth of 6 mm into the bilateral Zusanli acupoints (ST36), located 5 mm below and lateral to the anterior tubercle of the tibia [4, 12, 13]. ST36 acupuncture was performed immediately after closing the abdomen in the CLP procedure. EA stimulation was applied at both bilateral ST36 acupoints, and both output leads from the Programmable Electro-Acupuncture Stimulator (ITO ES 160 Electric Acupuncture Device, Tokyo, Japan) were connected to the handles of both needles inserted at ST36 acupoints. EA was applied for 20 min, with an intensity of 40 mA, a frequency of 30 Hz, and a 50 *μ*s pulse width. To control the effects of needle insertion, sham acupuncture was performed by needle stimulation of a nonacupoint that located the nearby ST36 in hamstring muscles. Rats that received stimulation of a nonacupoint were designated as “SHAM-EA”.

### 2.4. Subdiaphragmatic Vagotomy

Subdiaphragmatic vagotomy was performed on rats [14] anesthetized i.p. using ketamine (100 mg/kg) and xylazine (20 mg/kg). After the skin and abdominal wall were incised along the ventral midline (laparotomy), the stomach and lower esophagus were visualized and gently exposed in the abdominal cavity. For the complete vagotomy, the two ventral and dorsal trunks of the subdiaphragmatic vagus were identified on the esophagus, separated from the surrounding tissues under a dissecting microscope, and cut as high as possible on the esophagus below the diaphragm (1 cm above gastroesophageal junction). The neural and connective tissue surrounding the esophagus was removed to ensure transection of the small vagal branches. Sham animals were also prepared using a similar procedure, and the viscera were similarly handled, but no nerves were cut; the stomach was returned to its normal intra-abdominal position, and the laparotomy incision was closed in layers using a running suture of 3-0 sterile Nylon.

### 2.5. Catecholamine Depletion

Reserpine (Sigma-Aldrich, Saint Louis, MO, USA) was dissolved in DMSO (Sigma-Aldrich) and diluted in 96% ethanol (Sigma-Aldrich) to a concentration of 20% DMSO. The rats were administered reserpine at a dose of 10 mg/kg subcutaneously (s.c.) on the dorsum at 24 hrs before experimental CLP.

### 2.6. TNF and IL-6 Cytokines Measurement in Serum

The serum samples obtained from the rat groups were separated and stored at −70°C until thawing at the time of the assay. TNF and IL-6 were measured using highly sensitive enzyme-linked immunosorbent assay kits (Rat TNF and IL-6 ELISA Set, BD OptEIA, CA, USA) specific for rat cytokines according to the manufacturer's instructions.

### 2.7. Nitric Oxide (NO) Measurement in Serum

Nitrite was measured by addition of 100 *μ*L Griess reagent (21% sulphanilamide and 0.1% naphthalene diamine dihydrochloride in 5% phosphoric acid; Sigma-Aldrich) to 100 *μ*L of the serum. The absorbance at 540 nm was measured using a spectrophotometer (EPOCH Biotek, Winooski, VT, USA). The nitrite concentration was determined by using the standard concentrations of sodium nitrite (0–100 *μ*M).

### 2.8. HMGB1 Measurement in Serum

HMGB1 release was determined in rat serum using a specific anti-HMGB1 ELISA (IBL International, Hamburg, Germany) following the manufacturer's protocol.

### 2.9. Histological Analysis

The liver, kidneys, and lungs of the septic and sham-operated rats were harvested at 18 h after CLP. The tissue samples were fixed in 10% formalin solution, embedded in paraffin, and sectioned. The tissue sections were then stained with the hematoxylin and eosin reagent according to standard protocols and observed under light microscopy, and images were acquired with a Zeiss Primo Star microscope equipped with a camera (AxioCam ERc 5s).

### 2.10. Immunohistochemistry

After being dried for 45 minutes, paraffin sections were dewaxed in 2 changes of xylene for 15 and 20 minutes each, followed by a descending ethanol series and antigen retrieval in ethylene diamine tetra-acetic acid. The sections were incubated in 3% hydrogen peroxide for 15 min in a humidistat box at room temperature and rinsed in phosphate balanced solution (PBS) for 5 min⁡× 3. After incubating overnight at 4°C with polyclonal rabbit anti-rat NF-*κ*B p65 (1 : 1000; Santa Cruz Biotechnology, Santa Cruz, CA), the sections kept in the humidistat box were warmed to 37°C in an incubator for 45 minutes and then incubated with secondary antibodies (Immunohistochemistry Kit, Diagnostic BioSystems, Pleasanton, CA, USA) at 37°C for 30 min. After being washed with PBS for 5 min⁡× 3, the sections were made visible by using 3,3-diaminobenzidine, terminated on distilled water, and, subsequently, counterstained with hematoxylin for 1 minute. Finally, the slides were differentiated in 1% acid alcohol, blued in 1% ammonia water, dehydrated in graded concentrations of ethanol, cleared in 2 changes of xylene for 10 minutes each, and mounted with neutral gum. The sections were examined and photographed with a Zeiss Primo Star, light microscope at ×400.

### 2.11. Statistical Analysis

The data are expressed as means ± SEM. The statistical significance between specific groups was determined using one-way ANOVA with Bonferroni's post hoc test, and the statistically significant differences are indicated with asterisks: *P < 0.05 and **P < 0.001. The survival rates were compared using the Log-rank test, and the statistically significant differences are indicated with asterisks: *P < 0.05. All statistical analyses were performed using GraphPad Prism v5.0 (GraphPad Software, La Jolla, CA, USA).

## 3. Results

### 3.1. Electroacupuncture at the ST36 Acupoint Reduces Systemic Inflammation in a CLP-Induced Rat Model of Sepsis

**Figure 1:**
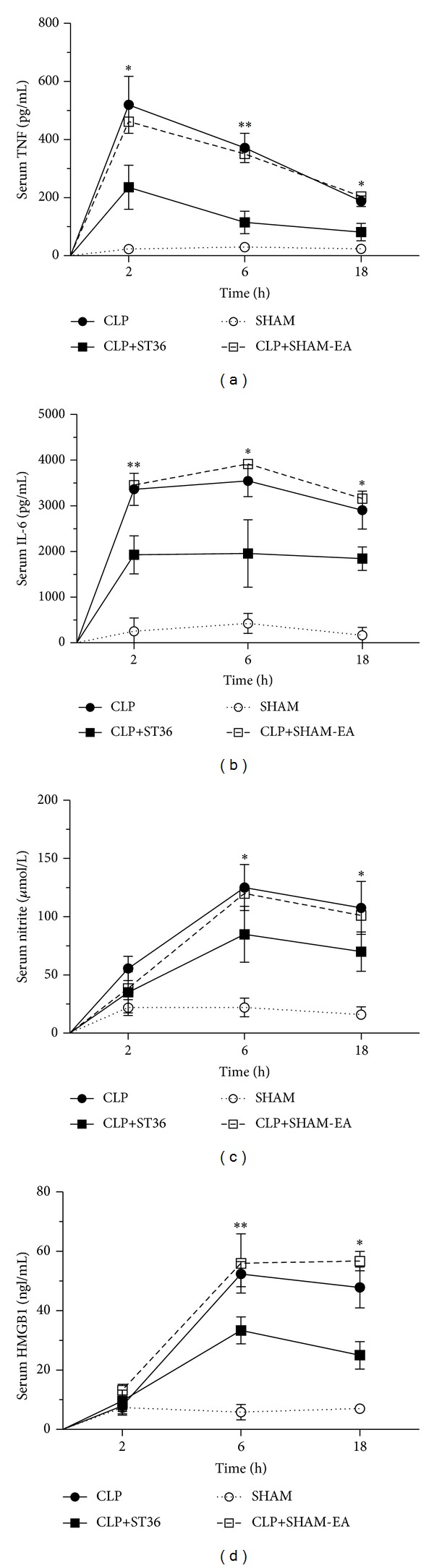
Effects of electroacupuncture on the serum TNF, IL-6, nitrite, and HMGB1 levels in a CLP-induced rat model of sepsis. Four groups of rats were formed: one group underwent surgery without CLP (SHAM) and three groups were subjected to CLP, where one of these groups was treated with EA at ST36 (CLP+ST36) or SHAM acupuncture (CLP+SHAM-EA) after closure and suturing of the abdominal cavity. The serum TNF (a), IL-6 (b), nitrite (c), and HMGB1 (d) levels were analyzed at 2, 6, and 18 hrs after the end of surgery in independent assay. The data are expressed as the means ± SD of 6 rats per group. The data were analyzed using one-way ANOVA and the Bonferroni test for multiple comparisons. Significant differences are indicated with an asterisk: *P < 0.05, **P < 0.01. The data are representative of three independent experiments.

To evaluate the effect of EA at ST36 on serum TNF, IL-6, nitrite, and HMGB1 levels during sepsis, four groups of rats were formed: one group underwent surgery without CLP (SHAM), and three groups were subjected to CLP, where one of these groups was treated with EA at ST36 (CLP+ST36) or sham acupuncture (CLP+SHAM-EA) after closure and suturing of the abdominal cavity. Stimulation was applied for 20 min, with an intensity of 40 mA, a frequency of 30 Hz, and a 50 *μ*s pulse width, at the ST36 acupoint to complete the CLP procedure. Blood samples were obtained at two, six, and eighteen hours after CLP to quantify the serum TNF, IL-6, nitrate, and HMGB1 levels in the groups. The rats treated with EA at ST36 showed lower serum TNF levels (means = 236.6 pg/mL; SD = 186.6) compared with the rats subjected to CLP alone (means = 519.8 pg/mL; SD = 239.6), and this difference was statistically significant (*P* < 0.05) (Figure 1(a)) at two hours after the stimulation, and the reduction lasted over 18 hrs. The rats treated with EA at ST36 showed lower serum IL-6 levels (means = 1 925.3 pg/mL; SD = 414.0) compared with the rats subjected to CLP alone (means = 3 358.6 pg/mL; SD = 701.8), and this difference was statistically significant (*P* < 0.01) at two hours after the stimulation, and the reduction lasted over 18 hrs (Figure 1(b)). EA at the ST36 acupoint in rats not subjected to CLP did not induce detectable serum TNF and IL-6 levels (data not shown).

EA also reduced serum nitrite levels at 6 and 18 hours after the stimulation (*P* < 0.05), but the inhibition was not statistically significant after 2 hrs (Figure 1(c)). Serum HMGB1 was decreased by 36.2% and 47.7% in the CLP+ST36 group compared with the CLP group at 6 and 18 hours after CLP, respectively (Figure 1(d)). These results indicate that electroacupuncture inhibited and did not merely delay mediators of inflammation production.

**Figure 2:**
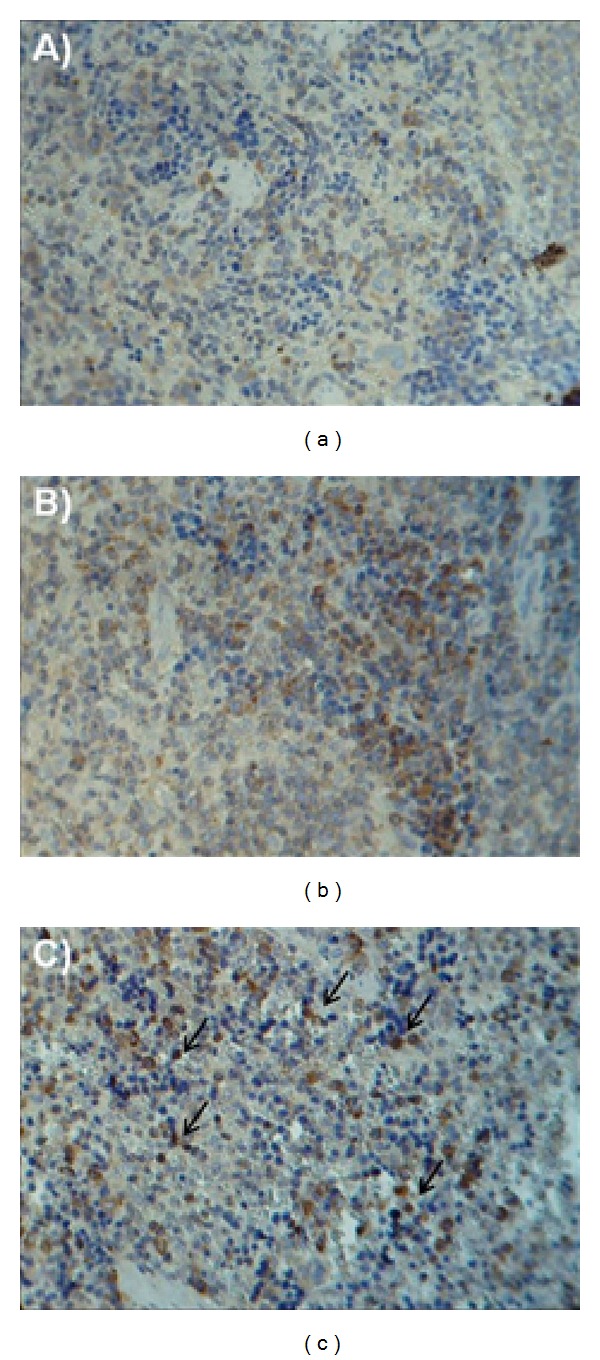
Effects of electroacupuncture on the translocation of NF-*κ*B in spleen in a CLP-induced rat model of sepsis. Three groups of rats were formed: one group underwent surgery without CLP (a), and two groups were subjected to CLP, where one of these groups was treated with EA at ST36 (b) or SHAM acupuncture (c) after closure and suturing of the abdominal cavity. Immunohistochemical staining of nuclear factor NF-*κ*B p65 in spleen from different groups at two hours after CLP. Original magnification: ×400.

Furthermore, immunohistochemistry was used to determine the nuclear fraction NF-*κ*B activity. The immunohistochemical staining of NF-*κ*B p65 of the SHAM group was distributed mainly in the cytoplasm with light brown coloration in the spleen at two hours after CLP (Figure 2(a)). However, the staining of NF-*κ*B p65 of rats subjected to CLP was dark brown in the nucleus (Figure 2(c)). On the other hand, in the group treated with EA, the staining became lighter and the nucleus could be clearly seen in blue (Figure 2(b)).

### 3.2. EA at ST36 Increases the Survival Rate in a CLP-Induced Rat Model of Sepsis and Ameliorates CLP-Induced Tissue Injuries to Liver, Kidneys, and Lungs

**Figure 3:**
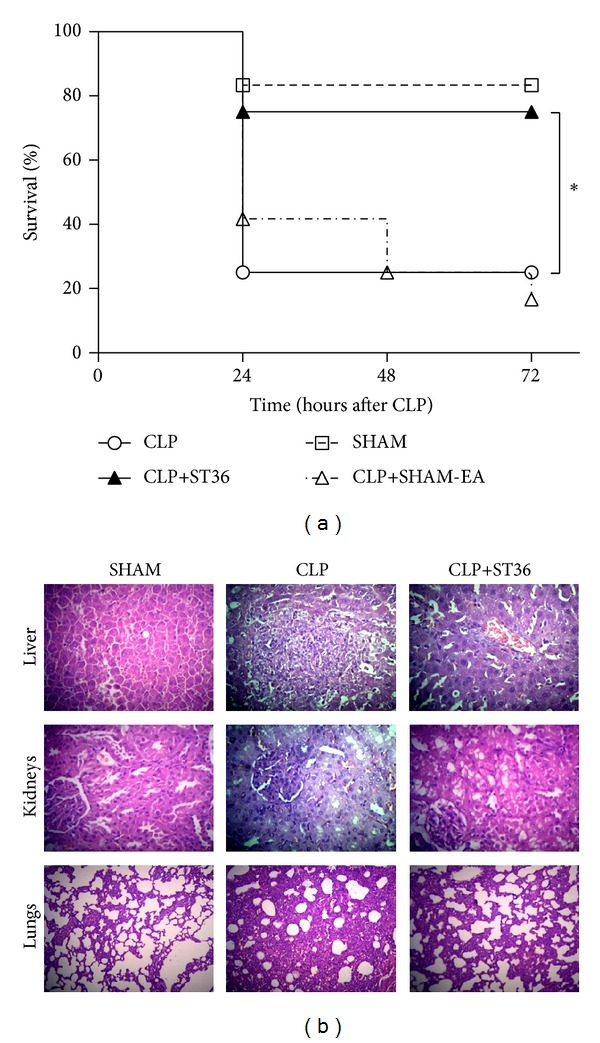
Effects of electroacupuncture on survival rates in a rat model of sepsis and organ injury induced by CLP. Four groups of rats (*n* = 12) were formed: one group underwent surgery without CLP (SHAM), and three groups were subjected to CLP, where one of these groups was treated with EA at ST36 (CLP+ST36) or SHAM acupuncture (CLP+SHAM-EA) after closure and suturing of the abdominal cavity. The percentage survival at 72 hours after surgery is shown. Differences in the survival curve were evaluated using the Mantel-Cox test. Significant differences are indicated with an asterisk: *P < 0.05. The data are representative of two independent experiments. (b) In independent assay, livers, kidneys, and lungs were harvested 18 h after CLP for histopathologic examination using hematoxylin and eosin staining. Representative images from six animals per group were shown. Original magnification: ×400.

To determine whether the reduced serum cytokines levels in a CLP-induced rat model of sepsis with EA at ST36 were associated with survival, four different groups of rats were used: one group underwent surgery without CLP (SHAM) and three groups were subjected to CLP (CLP), where one of these groups was treated with EA (CLP+ST36) or sham acupuncture (CLP+SHAM-EA) after closure and suturing of the abdominal cavity. The survival rate was evaluated within 72 hrs after surgery. The rats treated with EA showed 83.3% survival in contrast with the rats that were not subjected to EA, which showed a 25% survival, and this difference was statistically significant (*P* < 0.05) (Figure 3(a)).

Next, histopathological examinations of liver, kidneys, and lungs were used to determine the effect of EA-ST36 on CLP-induced organ injury. As shown in Figure 3(b), these morphological and acute inflammatory changes were attenuated in the group treated with EA at ST36 compared to the group not subjected to EA. The major acute inflammatory injuries in the liver from CLP-induced septic rats were extensive hepatic tissue malformation, intracellular and interstitial edema, and large area of necrosis. Those in the kidneys included interstitial inflammatory cell infiltration, kidney tubular hyperemia, endothelial cell swelling, and intercapillary cell proliferation. The major morphological alterations in CLP-induced lungs included the infiltration of leukocytes and leakage of erythrocytes into alveolar and interstitial spaces, edema, alveolar distortion, and thickening of the alveolar-capillary membrane. In contrast, rats treated with EA at ST36 showed minor liver, lungs, and kidneys damage. Altogether, EA treatment protects against acute organ injury induced by CLP.

### 3.3. The Reduction of the Serum TNF Levels and the Increased Survival Rate Induced after EA at ST36 Are Mediated through the Vagus Nerve in a Rat Model of Sepsis

**Figure 4:**
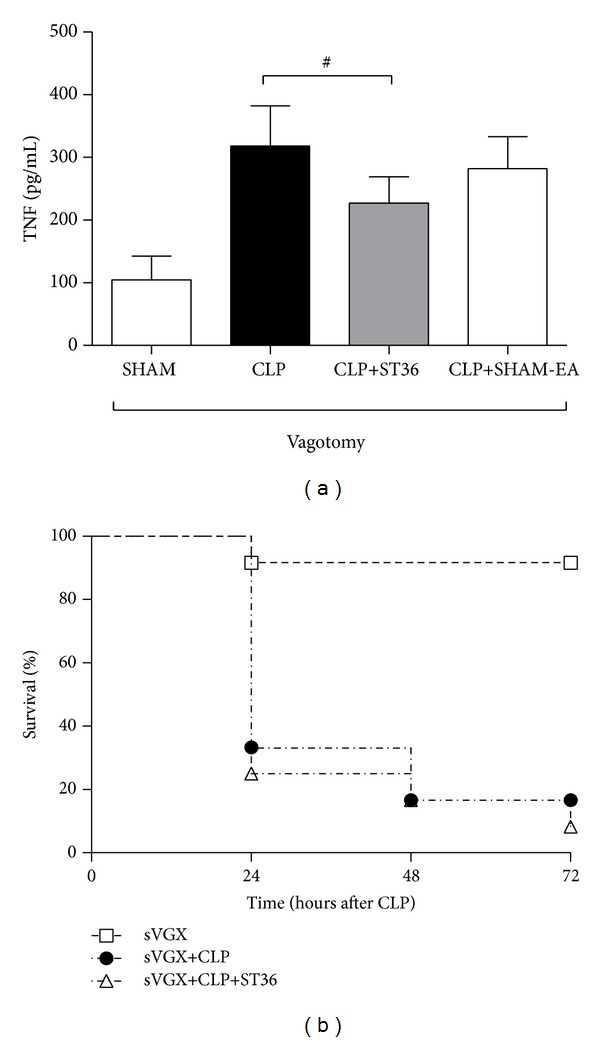
The reduction in the serum TNF levels after electroacupuncture in a rat model of sepsis is mediated through the vagus nerve and catecholamine production. Groups of six Wistar male rats with subdiaphragmatic vagotomy were subjected to experimental sepsis through cecal ligation and puncture (CLP) or only laparotomy without CLP (SHAM). Rats with CLP were subsequently treated with electroacupuncture at the ST36 acupoint or SHAM acupuncture (CLP+SHAM-EA), with an intensity of 40 mA, a frequency of 30 Hz, and a 50 *μ*s pulse width for 20 minutes (CLP+ST36). The serum TNF levels were analyzed at 2 hrs after surgery using ELISA (a). The data are expressed as the means ± SD of 6 rats per group. The data were analyzed using one-way ANOVA and the Bonferroni test for multiple comparisons. Results showing no significant differences are indicated with a numeric symbol. The percentage survival at 72 hours after surgery is shown (b). The data are representative of three independent experiments.

To determine whether the reduction in TNF expression observed in the CLP-induced model of sepsis after EA at ST36 is dependent on the cholinergic anti-inflammatory pathway (efferent VN) [14], rats were subjected to subdiaphragmatic vagotomy followed by surgical CLP. The rats treated with EA at ST36 showed no significant differences compared with the group of rats that were not treated with EA (*P* > 0.05). This result suggests that the reduction in the TNF levels after EA at ST36 during sepsis is dependent on the VN (Figure 4(a)). Furthermore, the survival rate was evaluated within 72 hours after surgery (Figure 4(b)) in independent groups of subdiaphragmatic vagotomy rats (sVGX); the group treated with EA did not show differences compared with the group of rats that were not treated with EA.

### 3.4. The Reduced Serum TNF Levels after EA in a Rat Model of Sepsis Are Mediated through Catecholamine Production

**Figure 5:**
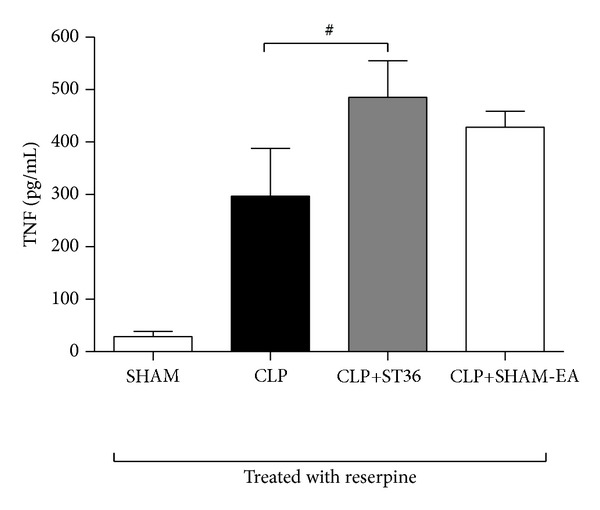
The reduction in the serum TNF levels after electroacupuncture in a rat model of sepsis is mediated through catecholamine production. Groups of six Wistar male rats were treated with a pharmacological inhibitor of catecholamines production (reserpine, 10 mg/kg sc) at 24 hrs before cecal ligation and puncture (CLP) or only laparotomy without CLP (SHAM). Rats with CLP were subsequently treated with electroacupuncture at the ST36 acupoint or SHAM acupuncture (CLP+SHAM-EA), with an intensity of 40 mA, a frequency of 30 Hz, and a 50 *μ*s pulse width for 20 minutes (CLP+ST36). The serum TNF levels were analyzed at 2 hrs after surgery using ELISA. The data are expressed as the means ± SD of 6 rats per group. The data were analyzed using one-way ANOVA and the Bonferroni test for multiple comparisons. The results showing no significant differences are indicated with a numeric symbol. The data are representative of three independent experiments.

A decrease in TNF production, mediated by the cholinergic anti-inflammatory pathway, has been suggested to occur following electrostimulation of the VN [9]. This decrease depends on the integrity of the VN and the expression of catecholamines. To determine whether the reduction of the serum TNF levels observed during sepsis after EA at ST36 is dependent on catecholamine, rats were pretreated with reserpine (an inhibitor of monoamine vesicular transport at presynaptic nerve neurons that inhibits the release of catecholamines in the synapse). The results showed that sepsis rats treated with reserpine and EA at ST36 did not show differences in the serum TNF levels compared with sepsis rats without EA treatment (Figure 5).

## 4. Discussion

Acupuncture has been widely applied for the treatment of inflammatory diseases [15]. Specifically, EA at the ST36 acupoint exerts an anti-inflammatory effect in animal models [3–6]. However, the underlying mechanisms and neural pathways associated with EA remain unknown. The findings reported in the present study indicate that EA induces an anti-inflammatory mechanism that reduces TNF, IL-6, nitrite, and HMGB1 production in one of the more widely used rat models for studying polymicrobial sepsis [11] (Figure 1). These results are consistent with previously reported data in a model of septic shock induced by LPS [16] and in a model of sepsis induced by CLP [17].

HMGB1 has previously been reported to be a cytokine that mediates organ damage in several sepses [18]. Previous studies demonstrated that a positive correlation was observed between HMGB1 and multiple organ system failure score in patients with septic shock [19]. Data from the present study suggests that an increased serum HMGB1 level is associated with damage of acute organ injury as shown by inflammatory histological alteration secondary to sepsis induced by CLP (Figure 3(b)).

Most of the harmful effects observed during sepsis have been ascribed to excessive production of inflammatory cytokines, such as TNF [20]. Consistent with these results, the highest serum TNF levels are observed at 2 hrs after CLP in a rat model of sepsis [21].

Previous studies have indicated that VN electric-stimulation during septic shock [16] and endotoxemia [14] specifically attenuates TNF production in spleen macrophages (main source of TNF in endotoxemia) through the cholinergic anti-inflammatory pathway [14], which is dependent on the anatomical and functional integrity of the VN, celiac-superior mesenteric plexus ganglia, and the splenic nerve [14]. The contribution of the VN was confirmed in the present study, as surgical vagotomy abolished the anti-inflammatory potential of electroacupuncture (Figure 4). The VN stimulation by EA-ST36 was verified by the decrease in heart rate variability (data not shown).

Some studies have suggested that the characteristics of the ST36 acupoint depend on the vagus nerve. Indeed, Torres-Rosas et al. reported that electroacupuncture at ST36 enhanced gastric myoelectric activity in rats, and this effect was VN dependent [17]. Peña et al. also reported a determinant role for N-methyl-D-aspartate receptors, which mediate synaptic transmission in gastric-projecting neurons of the dorsal motor nucleus of the VN, in the enhancement of gastric motility induced after stimulation at ST36 [22].

Still, other studies have reported that VN inhibits splenic TNF-*α* production through the activation of the splenic nerve to release norepinephrine [23]. Thus, it is reasonable to propose that a similar mechanism exists for electroacupuncture. Previous studies have indicated that norepinephrine inhibits TNF-*α* production in primary splenocytes via *β*2-adrenoceptors (*β*2AdrR) [22, 23]. The results obtained in the present study also suggest that the reduced TNF levels and increased survival rate in septic rats depend on the integrity of neurotransmission through catecholamine production and the anti-inflammatory effects of the ST36. The TNF levels are not reduced after catecholamine depletion through reserpine in septic rats, suggesting that these nerves are catecholaminergic and are required for the functional and pharmacological inhibition of TNF production through EA at ST36.

From a translational perspective, the anti-inflammatory potential of electroacupuncture prevents mortality in sepsis. Thus, future studies are warranted to determine the role of electroacupuncture in treating inflammatory conditions.

## 5. Conclusions

In conclusion, electroacupuncture at the ST36 acupoints reduced serum TNF, IL-6, nitrite, and HMGB1 levels and enhanced survival in septic rats. The reduced serum TNF level depends on VN integrity and catecholamine production. This approach could be developed as a treatment for sepsis or conditions associated with excessive inflammation.
